# Successful treatment of acute B lymphoblastic leukemia relapse in the skin and testicle by anti-CD19 CAR-T with IL-6 knocking down: a case report

**DOI:** 10.1186/s40364-020-00193-5

**Published:** 2020-05-06

**Authors:** Ze-Fa Liu, Li-Yun Chen, Jin Wang, Li-qing Kang, Hua Tang, Yao Zhou, Hai-Xia Zhou, Ai-Ning Sun, De-Pei Wu, Sheng-Li Xue

**Affiliations:** 1 Department of Hematology, People’s Hospital of Xinghua, Taizhou, Jiangsu Province China; 2 Jiangsu Institute of Hematology, The First Affiliated Hospital of Soochow University, Shizi street 188, Suzhou, 215006 China; 3 Institute of Blood and Marrow Transplantation, Collaborative Innovation Center of Hematology, Soochow University, Suzhou, China; 4 Faculty of Nursing, Suzhou Vocational Health College, Suzhou, China; 5 Shanghai Unicar-Therapy Biomed Phamaceutical Technology CO, LTD, Shanghai, China

**Keywords:** Acute lymphoblastic leukemia, Extramedullary relapse, Chimeric antigen receptor-modified T cell therapy, IL-6 knocking down

## Abstract

**Background:**

Extramedullary relapse is an important cause of treatment failure among patients with acute lymphoblastic leukemia (ALL). This type of relapse is commonly observed in the central nervous system, while it is rare in the testicles and skin. Chimeric antigen receptor-modified T cell (CAR-T) therapy targeting CD19 has shown to be a beneficial treatment approach for relapsed/refractory B cell acute lymphoblasticleukemia (r/r B-ALL). Yet, few studies have reported data regarding the treatment of extramedullary B-ALL relapse, especially both in skin and testicle, with CAR-T therapy.

**Case presentation:**

Here we reported a single case of a patient with relapsed B-ALL in skin and testicle who was successfully treated by the shRNA-IL6-modified anti-CD19 CAR-T(ssCAR-T-19) therapy. A 29-year-old man with relapsed B-ALL in skin and testicle was enrolled in clinal trial involving the shRNA-IL6-modified anti-CD19 CAR-T(ssCAR-T-19) therapy (ClinicalTrials.gov number, NCT03919240). The patient had toxicity consistent with the grade 1 cytokine release syndrome.

**Conclusions:**

ssCART-19 therapy may be used to effectively eliminate infiltrating leukemia cells in the skin and testicle with mild toxicity, which could be a much safer approach to bridge allo-HSCT, thus further improving the patient’s outcome.

**Trial registration:**

ClinicalTrials.gov number, NCT03919240, Registered 18 April 2019, retrospectively registered.

## Introduction

Extramedullary relapse, which is not a frequent recurrence of leukemia occurring in sites other than the bone marrow, is an important cause of treatment failure among patients with acute lymphoblastic leukemia (ALL) [1]. One-third of all extramedullary ALL relapses are reported in the central nervous system [2], while the relapses in testicle and skin are relatively uncommon [3, 4].

Chemotherapy is the first treatment of choice for patients with extramedullary ALL relapse followed by radiotherapy. Over the last few years, chimeric antigen receptor-modified T cell (CAR-T) therapy targeting CD19 has shown to be a beneficial treatment approach for relapsed/refractory B cell acute lymphoblastic leukemia (r/r B-ALL) [5, 6]. Yet, only very few studies have reported data regarding the treatment of extramedullary B-ALL relapse in skin and testicle with CAR-T therapy. Here we reported a single case of a patient with relapsed B-ALL isolatedly in skin and testicle with bone marrow remission who was successfully treated by the shRNA-IL6-modified anti-CD19 CAR-T (ssCAR-T-19) therapy. The feasibility and safety of a ssCAR-T-19 treatment are also discussed.

## Case presentation

A 29-year-old man, who presented with skin nodules and swollen testicle, was initially admitted to Shanghai Renji Hospital. Physical examination showed multiple red nodules in the skin and the swollen left testicle. Peripheral blood counts showed white blood cells total count of 7.0 × 10^9^/L, hemoglobin levels of 113 g/L, and platelets total count of 87 × 10^9^/L. Skin biopsy indicated B lymphocytic malignancies. Consequently, bone marrow aspiration analysis by morphology, immunophenotyping, cytogenetics, and molecular genetics suggested B-ALL. Moreover, the ultrasound examination confirmed the swollen left testicle. Finally, the patient was diagnosed with B-ALL, accompanied by extramedullary infiltration in the skin and testicle.

**Fig. 1:**
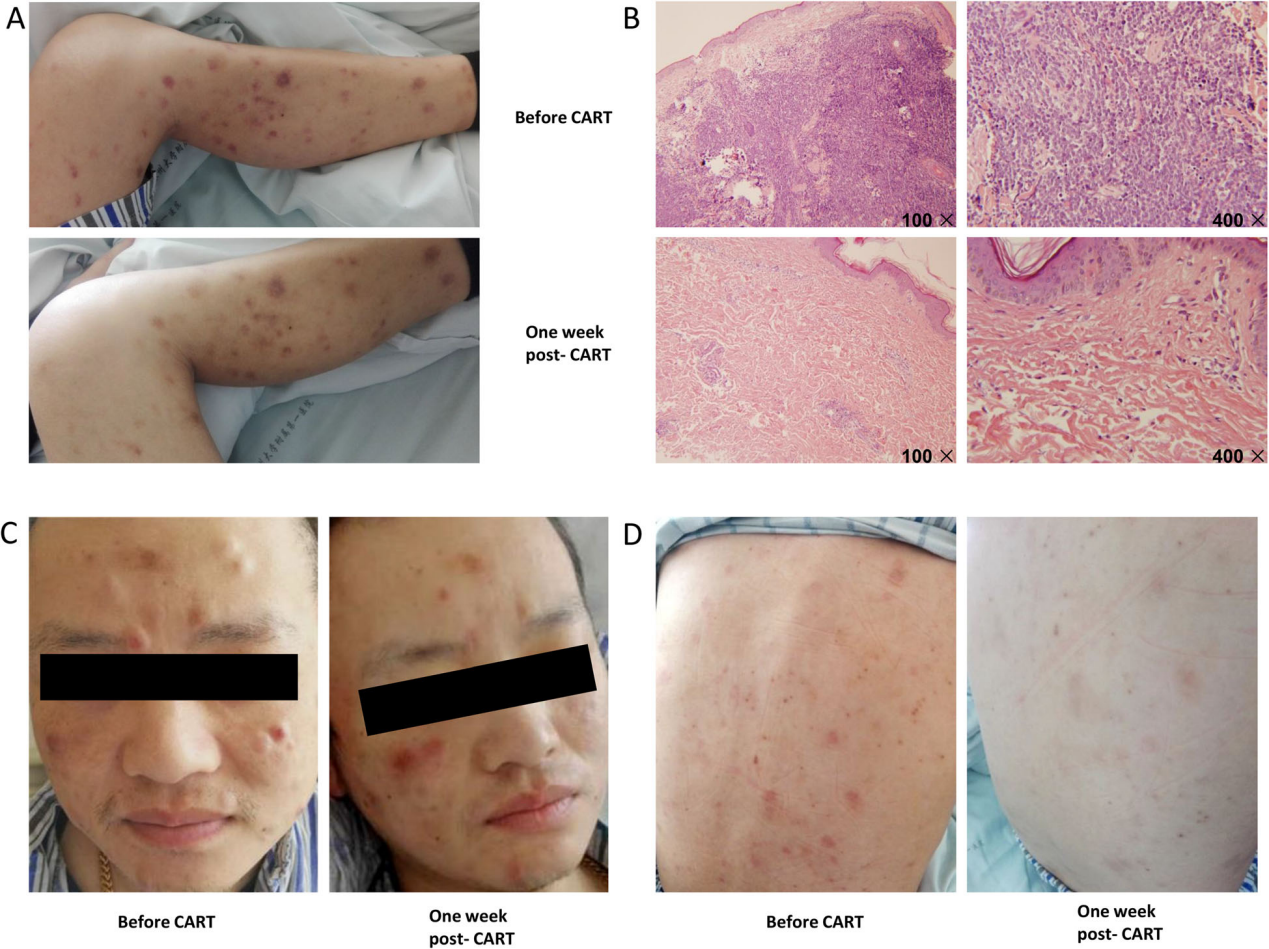
Changes in the patient’s skin before and after ssCART-19 cells infusion. **a**, Left lower limb skin. **b**, Skin biopsy from left lower limb and staining by HE. **c**, Facial skin. **d**, Back skin

**Fig. 2:**
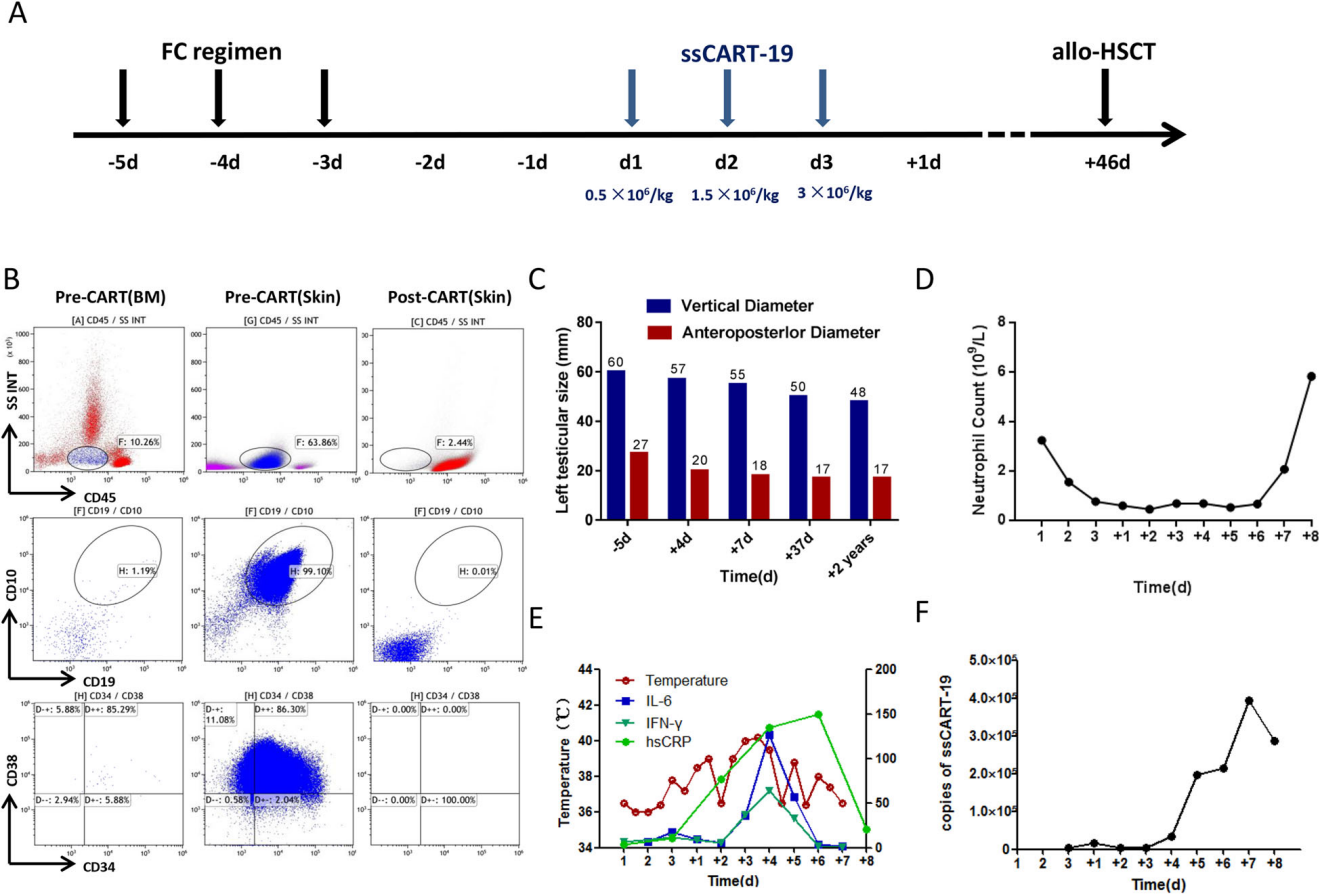
The summary of medication protocol, clinical and laboratory parameters relative to the timing of CART therapy. **a**, Chemotherapy for lymphpcyte depletion include fludarabine and cyclophosphamide. ssCART-19 cells were infused at a split-dose of 10% on day 01, 30% on day 02 and 60% on day 03(total 5 × 10^6^/kg). **b**, Expressions of blasts in bone marrow and skin were detected by flow cytometry before and after ssCART-19 treatment. **c**, Testiclar size was measured by ultrasound respectively on the -5 day (before CAR-T), on + 4 day (during CAR-T), + 7 day (after CAR-T), + 37 day (before HSCT) and most recently (after HSCT). **d**, Related hematological toxicity after ssCART-19 treatment. **e**, The trends of the patient’s temperature in degrees centigrade (°C) per 24-h period. IL-6(pg/ml), IFN(pg/ml) and hsCRP(mg/l) concentrations are shown in lines during CART therapy. **f**, The expansion levels of ssCART-19 cells in the peripheral blood (PB) were monitored by qPCR on each day

After induction therapy with VDLP (vincristine+daunorubicin+ L-asparaginase +prednisone), skin nodules disappeared, testes shrank, and complete remission was achieved, which was confirmed by bone marrow aspiration examination. However, after 3 cycles of consolidation treatment, the skin nodules (Fig. 1a, c, d) and the swelling of the scrotum reappeared (Fig. 2c).

Subsequently, the patient was referred to the hematology department of the First Affiliated Hospital of Soochow University. Bone marrow aspiration analysis by morphology, flow cytometry, and the next-generation sequencing showed no relapse. Flow cytometry analysis of skin biopsy showed 93.1% of blast proportion with immature B cell immunophenotyping profile (CD10+: 99.9%, CD22+: 99.8%, CD19+: 90.9%, CD20 + -: 97.9%, CD38+: 99%, CD34 + -: 73.9%)(Fig. 2b). The pathology of skin biopsy was consistent with B lymphoblastic lymphoma/ALL infiltration (Fig. 1b). Considering the poor responses in skin and testicle to conventional chemotherapy, the patient was enrolled in an anti-CD19 CAR-T clinical trial (ClinicalTrials.gov number, NCT03919240).

During the clinical trial, the T cells from the patient were purified from the blood by gradient centrifugation using Lymphoprep™ (Oriental Hua Hui, Beijing, China). Cells were then enriched using anti-CD3 magnetic beads (Miltenyi Biotec, Bergisch Gladbach, Germany). CD3 + T cells were stimulated with anti-CD3/CD28 (Miltenyi Biotec) monoclonal antibodies for 24 h and then transduced with recombinant lentiviral vectors encoding the CD19-specific CAR, and comprising an anti-CD19 single-chain variable fragment (scFv), 4-1BB costimulatory moiety, CD3-ζactivation domain, and IL-6 shRNA element, all manufactured by UniCar Therapy Ltd. CAR-T cells were cultured in AIM-V media (Gibco, Grand Island, NY, USA) supplemented with 10% autologous serum, 100 IU/ml IL-2 (Peprotech, Rocky Hill, USA), 5 ng/ml IL-7(Peprotech), and 5 ng/ml IL-15(Peprotech) for 12 days [7]. Before CAR-T cell infusion, the patient received lymphodepleting chemotherapy for 3 days with Fludarabine 30 mg/m^2^/day and Cyclophosphamide 250 mg/m^2^/day. Infection-related examinations including procalcitonin, spot test using T lymphocytes, 1, 3 - β glucan test, galactomannan test and chest CT scanning, were performed and showed negative results. Two days after conditioning, the patient received ssCART-19 cells infusion at a total dose of 5 × 10^6^ cells/kilogram for 3 consecutive days (Fig. 2a). Four days after infusion, the patient suffered from lower swelling in skin nodules and left testicle, accompanied by high fever. However, 1 week after infusion the patient’s skin nodules disappeared, and only the chromatosis remained (Fig. 1a, c, d). Biopsy showed few lymphocytes infiltration but no leukemia cells, which was confirmed by flow cytometry (Fig. 2b). Besides, the ultrasound examination revealed shirked testicles (Fig. 2c). Forty-six days after ssCART-19 therapy, the patient underwent allogeneic hematopoietic stem cell transplantation (allo-HSCT) from a matched unrelated donor, and at the moment is in complete remission. Until now he is followed up for 2 years and 1 month after HSCT with no signs of relapse in bone marrow, skin or testicles.

During the treatment, the indicators of cytokine release syndrome (CRS) and cytokine release encephalopathy syndrome (CRES) were comprehensively monitored. The levels of IL-6, IFN-γand hsCRP were elevated following ssCART-19 cells infusion; they were consistent with the changes in body temperature, and declined to basal level spontaneously in 1 week (Fig. 2e). CRS in grade 1 was considered only due to discomfort related to fevers (Fig. 2e) and no CRES was detected. The proliferation of ssCART-19 cells detected by the real-time quantitative PCR was noted on day + 6, and sustained on a high level for several days (Fig. 2f). The data of blood biochemical, BNP, troponin and D-dimer turned out to be normal. The patient had granulocytopenia without thrombocytopenia or anemia after infusion (Fig. 2d). The granulocytes recovered on day + 8.

## Discussion

The extramedullary ALL relapses in skin and testicle are rare. What’s more important, thus far there are no standard management protocols for patients with this rare isolated extramedullary relapse that may lead to dismal outcomes.

CAR-T therapy is a novel therapeutic strategy for r/r B-ALL patients [8–10]. CAR-T cells can migrate into the leukemia cell sanctuary, such as cerebrospinal fluid by overcoming the blood-brain barrier (BBB), which might be an important approach for solving the sanctuary leukemia problems with traditional chemical agents [11]. He et al reported anti-CD19 CAR-T as a feasible and safe treatment against central nervous system leukemia after intrathecal chemotherapy in adults with r/r B-ALL [12]. Furthermore, Dai and colleagues reported the beneficial effect of CAR-T infusion on patients with active CNSL [13]. All these data suggest that CAR-T cells can enter the central nervous system and eliminate leukemia cells. Thus, it is reasonable to postulate that CAR-T cells could use the same mechanism to target leukemia cells in other parts of the body, such as testicles and skin. Here we reported a single case with relapsed B-ALL in skin and testicle, which was successfully treated by the shRNA-IL6-modified anti-CD19 CAR-T (Fig. 2c).

Wang and colleagues reported that extramedullary ALL relapse in the cervix manifesting as a solid mass after allo-HSCT could be completely removed by CAR T-cell therapy [14], thus suggesting that CAR-T cells possess a highly penetrating and eradicating ability when encountering extramedullary leukemia mass. Due to a large number of capillaries, CAR-T cells should be able to infiltrate the skin and reach leukemia cells acting as subcutaneous nodules facilitated by the capillary transportation and working more efficiently, which was supported by the present case where skin nodules were significantly diminished (Fig.1a, c, d). Of note, a high level of CAR-T cells copies was detected in the peripheral blood during CAR-T treatment. This further demonstrated that vigorous CAR-T cells might eliminate extramedullary B-ALL relapse.

The severe CRS and CRES are still the main safety issues of CAR-T therapy in clinical management [15] caused by the rapid elevation of multiple cytokines, mainly IL-6 from activated CAR-T cells, and mononuclear cells, such as macrophage cells and DC cells. The activated CAR-T cells, stimulated by specific targeting cells, have an essential role in imitating CRS and CRES development. Since anti-IL-6 receptor monoclonal antibody, the standard procedure in the clinical management of severe CRS, could not penetrate BBB and limit the treatment of CRES, we generated a CAR construction with IL-6 shRNA. Our previous study (results not published) showed that the suppression of IL-6 gene expression in CAR-T cells significantly decreased the IL-6 release from monocyte in vitro, and potentially reduced the chance of severe CRS and CRES in vivo. Wang et al used conventional CAR-T cells to treat extramedullary relapse in the cervix and reported a low-grade CRS but severe CRES. Urinary incontinence was observed, which was likely due to neurotoxicity side effects from CAR-T therapy. Nevertheless, in our case, ssCART-19cells were applied, and no CRES was observed, thus suggesting that ssCART-19could be an alternative choice to avoid the side effects of conventional CAR-T therapy in special tissue such as CNS and testicle.

Unfortunately, the CAR-T cell copies were not detected in testicle and skin due to the ethical problem of taking the sample. So the infiltration efficiency of CAR-T cells from the blood into these extramedullary sites and proliferation dynamic in these sites remain unclear.

## Conclusions

ssCART-19 therapy may be used to effectively eliminate infiltrating leukemia cells in the skin and testicle with mild toxicity, which could be a much safer approach to bridge allo-HSCT, thus further improving the patient’s outcome.

## Data Availability

The datasets supporting the conclusions are included within this article.

## Abbreviations

ALL: Acute lymphoblastic leukemia
CAR-T: Chimeric antigen receptor-modified T cell
r/r B-ALL: relapsed/refractoryB cell acute lymphoblastic leukemia
ssCAR-T-19: shRNA-IL6-modified anti-CD19CAR-T
CRS: Cytokine release syndrome
CRES: Cytokine release encephalopathy syndrome
BBB: Blood-brain barrier
Allo-HSCT: Allologous hematopoietic stem cell transplantation
DC: Dendritic cells
CNS: Central nervous system
